# Predictors for recurrence of drug use among males on probation for methamphetamine use in Japan: a one-year follow-up study

**DOI:** 10.1016/j.dadr.2024.100316

**Published:** 2024-12-27

**Authors:** Ayumi Takano, Kunihiko Takahashi, Tatsuhiko Anzai, Takashi Usami, Shiori Tsutsumi, Yuka Kanazawa, Yousuke Kumakura, Toshihiko Matsumoto

**Affiliations:** a Department of Drug Dependence Research, National Institute of Mental Health, National Center of Neurology and Psychiatry, Tokyo, Japan; b Department of Biostatistics, M&D Data Science Center, Institute of Science Tokyo, Tokyo, Japan; c Graduate School of Health Management, Keio University, Kanagawa, Japan; d National Hospital Organization, Hizen Psychiatric Center, Saga, Japan; e Department of Neuropsychiatry, The University of Tokyo Hospital, Tokyo, Japan

**Keywords:** Methamphetamine, Recurrence of drug use, Community-based program, Criminal justice system, Cohort study

## Abstract

**Background:**

Methamphetamine use is related to severe health, social, and criminal challenges. However, there is limited evidence regarding the factors associated with the recurrence of drug use among individuals who have used methamphetamine, particularly within populations involved in the criminal justice system. This study aimed to identify predictors of illicit drug use at a one-year follow-up among males in Japan who have used methamphetamine and are involved in the criminal justice system.

**Methods:**

The study participants were adult males on probation due to methamphetamine use or possession and were involved in a community-based program. The participants were recruited early in their probation period and participated in telephone-based surveys conducted by mental health center staff. We analyzed one-year follow-up data to investigate the recurrence rate of illicit drug use and associated risk factors using multiple logistic regression.

**Results:**

Out of 234 participants, 27 (11.5 %) used illicit drugs during the one-year follow-up period. After adjusting for demographic characteristics, severity of drug use, type of probation, and use of treatment for substance use disorders, the use of social welfare services (OR = 2.78) and a lack of trustworthy relationships (OR = 3.17) were significantly associated with recurrence of illicit drug use.

**Conclusions:**

This study suggested that individuals facing challenges in maintaining stable living conditions and building trustworthy relationships were more likely to return to drug use early in their probation period. Comprehensive and tailored support focused on social stabilization and relationship-building is recommended to aid recovery in males who have experienced methamphetamine use.

## Highlights

• Few studies on community-based programs for individuals who use drugs.
• Predictors for recurrence are unclear among individuals who use methamphetamine.
• One-year prospective cohort study among Japanese males who use methamphetamine.
• Unstable life and social isolation are risk factors for recurrence of drug use.

## 1 Introduction

Methamphetamine use is increasingly recognized as a public health concern globally, and the quantity of seized methamphetamine has also increased by 3 % from 2020 to 2021 (Degenhardt and Hall, 2012, McDonell and Rawson, 2023, United Nations Office on Drugs and Crime, 2023). Methamphetamine use is related to drug dependence, mental health issues, blood-borne and sexually transmitted infection diseases, and cardiac effects (Huang et al., 2023, Manja et al., 2023, McKetin et al., 2016, Vu et al., 2016). The concern of disease from amphetamine/methamphetamine use presents significant challenges in many countries with distinct geographic patterns. In Asia, amphetamine dependence accounts for 52 % of disability-adjusted life years attributed to amphetamine use disorders (Whiteford et al., 2013). Particularly in the East and South East Asia region, the population who use methamphetamine has increased, and the primary drug reported by individuals in drug treatment is methamphetamine (United Nations Office on Drugs and Crime, 2023). Similar to other Asian countries, in Japan, methamphetamine is the most prevalent drug in drug dependence treatment and the criminal justice system (Kondo et al., 2023, Research and Training Institute of the Ministry of Justice, 2023, Yamamoto et al., 2023).

Methamphetamine use is also related to social and criminal challenges, including hospitalization, incarceration, lost productivity, reduced income, disruption of family life, and social adversity (Guerin et al., 2023, Marshall and Werb, 2010, Sampson et al., 2023, Shoptaw et al., 2022, Sommers et al., 2006). Economic costs attributed to methamphetamine use have a notable impact on the criminal justice system (Cumming et al., 2020, Dobkin and Nicosia, 2009, Guerin et al., 2023, Tait et al., 2018). In the US, a previous study reported that half to two-thirds of incarcerated individuals had substance use disorder (Graves and Fendrich, 2024). Additionally, most formerly incarcerated individuals reentering the community were likely to be rearrested for a crime within a few years after release (Graves and Fendrich, 2024, Hazama and Katsuta, 2023). Accordingly, preventing the recurrence of drug use is essential not only for the health and well-being of individuals with a history of convictions related to methamphetamine use but also for public health and to alleviate the economic concern on society.

Although intervention studies in clinical settings have revealed the effectiveness of intensive treatment for people with methamphetamine use disorder, few studies have examined the efficacy of community-based programs in real-world settings. Additionally, in Japan, some forms of community-based support are available for individuals who use drugs. However, no studies have been conducted to evaluate the characteristics of participants or the effectiveness of these programs. A recent scoping review on community-based drug use treatment programs for reentering society among justice-involved individuals found inconclusive results regarding the effectiveness of these programs on substance use outcomes (e.g., days of drug use, abstinence) and criminal justice outcomes (e.g., recurrence of drug use, reinvolvement with the justice system, days in jail) (Graves and Fendrich, 2024). Additionally, most studies on these programs were conducted in the U.S., and intervention methods varied by study (e.g., peer support, case management, cognitive behavioral therapy, contingency management, and mindfulness program) (Graves and Fendrich, 2024). The primary drug focus was generally non-specific or involved multiple substances; however, when studies targeted specific substances, medication for opioid use disorder was most prominent (Graves and Fendrich, 2024). Therefore, there is limited evidence regarding community-based programs targeting individuals with a history of methamphetamine use, particularly in regions where methamphetamine is highly prevalent in the criminal justice system.

Various complex individual, social, and environmental factors are considered to influence the recurrence of drug use or recovery from methamphetamine use, as well as the capacity to engage in and benefit from preventive support and treatment (Brookfield et al., 2019, O’Donnell et al., 2019). In a systematic review and thematic synthesis of qualitative studies on factors related to the recurrence of methamphetamine use by O'Donnell et al. (2019), individual factors included several critical events (e.g., unemployment, loss of relationships), lack of social support, persistent mental health issues and trauma, and coping with stress through methamphetamine use. Social factors included social pressures, work instability, demands for long working hours and high performance, and association with former drug-using networks. Environmental factors included ecological stressors (e.g., unstable housing, food insecurity, and financial instability) and welfare deprivation. On the other hand, individual factors related to recovery from methamphetamine use included awareness of physical and mental health concerns, personal qualities (e.g., willpower, self-awareness), and experiences of the negative effects of drug use. Social factors included support from family or friends, associations with non-drug-using peers, changes in social networks, and securing lawful employment. For environmental factors, it was unclear whether the effects of imprisonment were sustained after release from jail (O’Donnell et al., 2019, Hazama and Katsuta, 2023).

There is limited empirical research on community-based programs for individuals who use methamphetamine and the predictors associated with the recurrence of drug use. This study aimed to investigate the risk factors associated with the recurrence of drug use among individuals involved in Japan's criminal justice system due to methamphetamine use, who also participated in a community-based support program.

## 2 Methods

### 2.1 Survey procedure and participants

We used data from a multicenter prospective cohort study called the "Voice Bridges Project (VBP)." The VBP is a research-based project started in 2017 to collect data from individuals on probation due to drug use or possession in Japan and establish a system to support their reentry into the community (Matsumoto, 2022, Matsumoto, 2019). The inclusion criteria for the VBP were: 1) aged 20 or older, 2) residing in areas covered by public mental health centers participating in VBP, 3) on probation for controlled drug use or possession (e.g., methamphetamine, cannabis), and 4) agreed to participate in the VBP. In the Japanese criminal justice system, most individuals arrested for drug use or possession for the first time typically receive a suspended sentence without probation or imprisonment. Thus, the VBP participants had been arrested more than twice because they resumed using drugs shortly after the first sentence. Potential candidates for the VBP were voluntarily recruited at the probation office and referred to a public mental health center. Public mental health centers in Japan are centralized institutions that provide mental health treatment services, including for substance use issues, as well as welfare support for individuals seeking assistance. Healthcare workers at public mental health centers, including public health nurses, psychiatric social workers, or clinical psychologists, obtained document-based consent from the participants and conducted an in-person baseline survey. Telephone-based surveys were conducted starting from the second follow-up survey, and the cohort study lasted for three years. In the first year, there were four surveys, each conducted every three months, and in the second and third years, two surveys were conducted every six months. The staff at each mental health center entered survey data, including confidential participant information, into a database specifically developed for the VBP.

Although the primary purpose of the VBP was data collection, the telephone-based surveys also served as a form of low-intensity support. During telephone-based surveys, mental health center staff often additionally asked participants about health conditions and healthcare needs. Based on participants’ needs, they provided support, such as telephone consultation, referrals to private rehabilitation facilities or treatment for substance use disorder, and assistance with accessing welfare services. However, this support was optional, and the types of support varied for each individual. Some participants received support, while others did not receive any. During their probation period, the participants received regular supervision from probation officers and underwent drug tests. If a drug test was positive, the participant was typically incarcerated immediately. However, if the participant disclosed illegal drug use to mental health center staff during the follow-up surveys, the information was not reported to probation officers. The confidentiality of study data was explained to participants as part of the informed consent process.

From 2017 to December 2022, the number of mental health centers participating in the VBP gradually increased to 23 out of 69 in Japan (Matsumoto, 2022). By December 2022, a total of 753 individuals participated in the VBP, with 319 (response rate: 79.2 %), 182 (78.4 %), and 98 (75.4 %) participants completing the one-year, two-year, and three-year follow-up surveys, respectively (Matsumoto, 2022). The participation rate among all VBP candidates was about 11 % (Matsumoto, 2019, Matsumoto, 2017). In this study, we focused on the majority of participants (male individuals) because the number of female participants in the VBP was small, and females who use drugs often have different backgrounds, such as initiation of drug use or support needs for reentry into the community. We selected participants based on the following criteria: 1) males on probation due to methamphetamine use or possession, 2) completed the one-year follow-up survey by December 2022, and 3) no missing variables for the analyses.

### 2.2 Measures

#### 2.2.1 Outcome variable

The dependent variable was defined as any illicit drug use at least once between the baseline and the one-year follow-up survey, categorized as a binary variable (yes or no). Illicit drugs included all illegal substances in Japan, such as methamphetamine, other amphetamine-type stimulants, cocaine, cannabis, heroin, and new psychoactive drugs. Drug use was self-reported during a telephone survey.

#### 2.2.2 Independent variables

We used the following independent variables, which were considered potential risk factors for the recurrence of drug use.

##### 2.2.2.1 Severity of drug use issues

The severity of drug use issues was assessed using the Drug Abuse Screening Test-20 (DAST-20) (Skinner, 1982). The DAST-20 is a self-reported scale in which higher scores indicate more severe drug use issues. The reliability and validity of the Japanese version of the DAST-20 has been confirmed (Shimane et al., 2015). The DAST-20 asked about drug use issues in the past 12 months; however, most participants could not use drugs because of incarceration. Thus, in this study, participants responded to the DAST-20 based on their status 12 months before their arrest. We also collected data on the age of drug use onset, duration of drug use (calculated as current age minus age of onset), and the number of arrests under the *Drug Control Act*.

##### 2.2.2.2 Probation type

In the Japanese criminal justice system, there are three types of probation for adults convicted of drug-related crimes: partial stay of execution of sentence, parole, and whole probation without imprisonment. Probation orders, except for probation without imprisonment, are implemented following a period of imprisonment. The system of parole with partial suspension of execution was introduced in June 2016 to reduce recidivism and support reentry into the community, aiming to avoid lengthy incarceration for individuals repeatedly arrested due to drug use (Kato, 2018). Generally, individuals ordered a partial stay of execution of sentence are required to undergo a longer probation period (ranging from one to five years) instead of serving a prison sentence, compared to those with other types of probation. In contrast, individuals on parole typically serve probation for some months. The length of probation might influence the recurrence of drug use and reentry into the community because individuals on probation receive regular drug tests and supervision by probation officers. Therefore, we categorized the variable of probation type into a binary variable (partial stay of execution of sentence or no partial stay).

##### 2.2.2.3 Use of social welfare and treatment for substance use disorder

Information on using social welfare services (such as public assistance and social security benefits) at baseline was categorized as a binary variable (yes or no). Additionally, we assessed the use of substance use disorder (SUD) treatment programs provided by medical facilities, public mental health centers, probation offices, rehabilitation facilities, and 12-step-based programs. We categorized it as a binary variable (yes or no).

##### 2.2.2.4 Interpersonal relationships

We inquired about the participants' trustworthy interpersonal relationships by asking, "Do you have someone with whom you can talk openly about anything, including drug use?" Responses were classified as "no one" and "yes, at least one person."

##### 2.2.2.5 Sociodemographic variables

At the baseline survey, we assessed age, job status (unemployed or employed), education attainment (junior high school, high school, or bachelor's degree or higher), and marital status (unmarried or married).

### 2.3 Statistical analysis

First, we calculated the recurrence rate of illicit drug use and described the participants' characteristics by recurrence status (abstinence group vs. recurrence group). Next, simple logistic regression was used to compare participants' characteristics at the baseline. Finally, multiple logistic regression was performed to assess the association between potential predictors and the recurrence of illicit drug use. We excluded an independent variable if a strong correlation (|r| > 0.7) was observed between each other. To confirm the robustness of the results, sensitivity analysis was performed, excluding independent variables with a medium correlation. All statistical analyses were performed using Stata 16 for Windows (StataCorp L.P., College Station, TX). Statistical significance was set at 0.05, and all tests were two-tailed.

### 2.4 Ethical considerations

This study was approved by the Institutional Review Board of the National Center of Neurology and Psychiatry (No. A2016–088). The original data, including the participants' private information, was protected from access by other institutions. The study data was strictly stored, and the staff and researchers were required to provide an ID and password when accessing the database. The researchers downloaded data without private information from the database for analysis.

## 3 Results

Of 319 participants who completed a one-year follow-up survey, data from 234 (73.4 %) participants were analyzed after excluding females (n = 56), individuals on probation for drug use or possession of substances other than methamphetamine (n = 2), and cases with missing variables required for analysis (n = 27). Among 234 participants, 27 (11.5 %) reported illicit drugs by the one-year follow-up. Table 1 shows participants' characteristics at the baseline between the abstinence and recurrence groups. Participants' mean age was 45.5 years (standard deviation [SD] = 10.2), and the average onset age of drug use was 20.2 (SD = 7.1). Drug issue severity assessed by DAST-20 was 11.4 (SD = 3.5), which meant moderate to severe drug dependence. The recurrence group was predominantly unmarried (96.3 %), whereas 76.8 % of the abstinence group were unmarried. Additionally, 29.6 % of individuals in the recurrence group lacked trustworthy interpersonal relationships, while this was for 15.5 % of the abstinence group.

**Table 1:** Participants' characteristics by recurrence status (N = 234).

		Abstinence (n = 207)			Recurrence (n = 27)	
		N/Mean	%/SD	N/Mean		%/SD
Age (mean/SD)		46.2	9.5	44.9		11.0
Job status	Unemployed	100	48.3 %	12		44.4 %
	Employed	107	51.7 %	15		55.6 %
Educational attainment	Junior high school	73	35.3 %	7		25.9 %
	High School	100	48.3 %	16		59.3 %
	Bachelor's degree or higher	34	16.4 %	4		14.8 %
Marital status	Unmarried	159	76.8 %	26		96.3 %
	Currently married	48	23.2 %	1		3.7 %
Social welfare service use	No	158	76.3 %	17		63.0 %
	Yes	49	23.7 %	10		37.0 %
SUD treatment or support service use	No	47	22.7 %	8		29.6 %
	Yes	160	77.3 %	19		70.4 %
Probation type: Partial stay of execution of sentence	No	123	59.4 %	20		74.1 %
	Yes	84	40.6 %	7		25.9 %
Trustworthy interpersonal relationship^a^	Yes, at least one person	175	84.5 %	19		70.4 %
	No one	32	15.5 %	8		29.6 %
Onset age of drug use (Mean/SD)		20.4	7.2	20.0		7.1
Duration of drug use (Mean/SD)		25.8	10.5	24.9		9.6
Drug issue severity: DAST−20^b^ (Mean/SD)		10.8	3.9	11.9		3.1
Number of arrests under the Drug Control Act (Mean/SD)		2.4	2.1	2.5		2.0

a : "Do you have a person with whom you can talk openly about anything, including drug use?"

b : DAST-20: Drug abuse screening test 20

Table 2 presents factors associated with the recurrence of illicit drug use using simple logistic regression or multiple logistic regression. When conducting multiple logistic regression, we excluded age because age was correlated with duration of drug use (r = 0.75, p < 0.001) and number of arrests (r = 0.40, p < 0.001; Supplement Table 1). Additionally, we excluded the variable of marital status because only one individual in the recurrence group was married (Table 1). As a result of multiple logistic regression, participants who utilized social welfare services had significantly higher odds of drug use compared to participants who did not (odds ratio [OR] = 2.78, p = 0.032, 95 % confidence interval [CI]: 1.09–7.05). Those without trustworthy interpersonal relationships had significantly higher odds of drug use compared to those with such relationships (OR = 3.17, p = 0.023, 95 % CI: 1.17–8.60). Job status, educational attainment, SUD treatment or support service use, probation type, duration of drug use, and drug issue severity were not significantly associated with the recurrence of drug use.

**Table 2:** Factors associated with recurrence of illicit drug use: results of simple logistic regression and multiple logistic regression analysis (N = 234).

		Non-adjusted				Adjusted			
		OR	95 % CI		p	OR	95 % CI		p
Job status	Employed	ref.				ref.			
	Unemployed	1.17	0.52	2.62	0.706	1.56	0.63	3.86	0.336
Educational attainment	Junior high school	ref.				ref.			
	High School	1.67	0.65	4.26	0.285	1.86	0.67	5.17	0.234
	Bachelor's degree or higher	1.23	0.34	4.48	0.757	1.33	0.34	5.21	0.684
Social welfare service use	No	ref.				ref.			
	Yes	1.90	0.82	4.41	0.137	**2.78**	**1.09**	**7.05**	**0.032**
SUD treatment or support service use	No	ref.				ref.			
	Yes	0.70	0.29	1.70	0.427	0.81	0.29	2.24	0.684
Probation type: Partial stay of execution of sentence	No	ref.				ref.			
	Yes	0.51	0.21	1.27	0.147	0.57	0.21	1.5	0.268
Trustworthy interpersonal relationship^a^	Yes, at least one person	ref.				ref.			
	No one	2.30	0.93	5.71	0.072	**3.17**	**1.17**	**8.60**	**0.023**
Duration of drug use		0.99	0.95	1.03	0.676	0.98	0.94	1.03	0.496
Drug issue severity: DAST−20^b^		1.08	0.97	1.21	0.153	1.10	0.97	1.23	0.132
Number of arrests under the Drug Control Act		1.02	0.84	1.23	0.868	0.98	0.76	1.25	0.847

a : "Do you have a person with whom you can talk openly about anything, including drug use?"

b : DAST-20: Drug abuse screening test 20

For sensitivity analysis, we excluded the variable of duration of drug use because it was correlated with the onset age of drug use (r = -0.44, p < 0.001) and the number of arrests (r = 0.48, p < 0.001; Supplement Table 1). Even after excluding the duration of drug use, the results of multiple logistic regression remained consistent with the main findings (Supplement Table 2).

## 4 Discussion

This study investigated the recurrence rate of illicit drug use and its associated risk factors among males on probation for methamphetamine use or possession who participated in VBP, which was a community-based program in Japan. The participants received telephone-based follow-up surveys and low-intensity support tailored to their individual needs. By the one-year follow-up, approximately one in ten participants had used illicit drugs again. The use of social welfare services and a lack of trustworthy interpersonal relationships were identified as risk factors for the recurrence of illicit drug use.

The participants in this study were males on probation for methamphetamine use or possession who also completed telephone-based one-year follow-up surveys conducted by public mental health center staff. The response rate was relatively high at approximately 80 %. However, a previous report documented that only 11 % of eligible participants agreed to enroll in the cohort study. Differences in characteristics were observed between VBP participants and non-participants (Matsumoto, 2019). Among VBP participants, a higher proportion of individuals were sentenced to partial stay of execution of sentence as probation status (the VBP participants: 24.9 %, non-participants: 16.4 %). Additionally, VBP participants were less likely to be arrested by the end of their probation period (Matsumoto, 2019). A low participant rate is not unexpected among individuals who use drugs and are involved in the criminal justice system, as participation in this study was entirely voluntary and offered no incentives. However, the findings in this study may have been influenced by selection bias.

Although it is difficult to compare this study with other studies because of different criminal justice systems across countries and variations in participant characteristics, the one-year recurrence rate of illegal drug use observed in this study (11.5 %) was relatively low. For example, a study in Taiwan among individuals who used methamphetamine and participated in joint legal-medical treatment programs as a diversion intervention reported a methamphetamine use rate of 37.8 % during a 12-month treatment period (Huang et al., 2023). In our study, participants were recruited voluntarily, separating them from the criminal justice system. Additionally, the mental health center staff regularly contacted the participants during the confidential follow-up surveys and checked on their support needs. This approach likely enabled participants to access help more easily without fear of mandatory penalties. As a result, participants may have been able to maintain abstinence, similar to the outcomes seen in other community-based programs for criminal justice-involved individuals in other countries (Graves and Fendrich, 2024).

In terms of risk factors associated with the recurrence of illicit drug use, the results in this study suggested the use of social welfare services, including public assistance and social security benefits, as well as lack of trustworthy interpersonal relationships, even after adjusting for the severity of drug issues and socioeconomic status. While previous studies have identified　various potential risk factors associated with clinical outcomes, our findings emphasize that social vulnerability and social isolation were significant predictors for the recurrence of drug use. On the other hand, severe drug craving and methamphetamine use at baseline predicted a shorter length of time to the recurrence in the Taiwan study (Huang et al., 2023). One possible explanation for these differences is the variation in phases of drug use. Scientific literature defines distinct phases of methamphetamine use including initiation, continuation/progression, increase/uncontrolled use, and decrease/cessation. This suggests the necessity of providing tailored interventions based on the specific phase of methamphetamine use because individuals may develop different patterns of drug use, experience varying drug-related harms, and have unique needs throughout their life course (Boeri et al., 2009, Liebregts et al., 2022, Martens et al., 2020, O’Donnell et al., 2019). Especially during the phase of decrease or cessation, various social and environmental factors are considered essential in addition to individual health factors (Liebregts et al., 2022, O’Donnell et al., 2019). For example, relationships with family members and non-drug-using friends, changing or distancing themselves from particular social networks, stable housing, financial security, and licit employment can support or encourage abstinence and reduce methamphetamine use (O’Donnell et al., 2019). Additionally, prolonged abstinence from methamphetamine is considered a protective factor for individual clinical conditions such as decision-making, emotional state, and craving among patients with methamphetamine use disorder (Wang et al., 2013). The participants in this study had a certain period of abstinence because of imprisonment or detention, suggesting they were likely in the decrease/cessation phase. Therefore, social and environmental factors may have played a greater role in the recurrence of drug use rather than the severity of individual drug issues. To promote reentry into the community, it is crucial to provide support not only for accessing treatment for substance use disorder but also for establishing a stable and secure life, as well as fostering trustworthy interpersonal relationships for criminal justice-involved males.

The concept of social recovery has been introduced in the field of drug use issues, which directs attention to the social environment with an emphasis on human interaction. It refers to the process of acquiring the skills, resources, and networks needed to help individuals live in society without resorting to substance use (Boeri et al., 2014, Boeri et al., 2011, Boshears et al., 2011). Social recovery emphasizes the development of social interactions, the importance of social bonding, and bridging relationships, even if individuals do not completely stop using drugs (Boeri et al., 2014). A strong stigma surrounding methamphetamine use and imprisonment is deeply rooted in Japan. This interferes with re-employment among individuals who use drugs, access to rental housing, and rebuilding trust with former social connections. Individuals who face challenges in getting a stable income or living a secure life may be more likely to rely on social welfare services, which can cause the recurrence of drug use. Additionally, stigma or punishment approaches may result in the loss of social connections and interactions with others. Therefore, interventions employing the concept of social recovery may be beneficial for males with methamphetamine convictions to prevent social isolation, an unstable social life, the recurrence of drug use, and to reintegrate into the community.

We acknowledge some limitations in our study. First, we included only males on probation who completed the one-year follow-up survey (response rate: 79.2 %). As a result, the findings in this study may not be generalizable to individuals who discontinued the cohort study earlier. Those who discontinued the cohort study by the one-year follow-up (approximately 20 % of participants) may not have responded to the survey due to their drug use, which could lead to an underestimation of the recurrence of drug use in this study. Furthermore, the results are not adaptable for long-term outcomes beyond one year. Second, we did not adjust all confounders highlighted in previous studies, such as mental health conditions and psychological distress. Third, the outcome measure of illicit drug use was self-reported and may have been influenced by social desirability bias. Although participants were assured that their reports of drug use would not be shared with the probation officers, the recurrence rate might be underestimated. Finally, the accuracy of estimates may be low with wide 95 % confidence intervals, likely due to the small sample size and small number of recurrences. Longer-term longitudinal studies with a larger sample size and adjustments for individual health conditions are needed to strengthen the findings.

## 5 Conclusions

Individuals who resumed drug use within one year of imprisonment or detention likely encountered challenges in achieving a stable life and reforming trustworthy relationships, which causing their recurrence of drug use. Comprehensive, tailored support that prioritizes social and economic stabilization as well as relationship-building is essential to promote recovery in males with a history of methamphetamine use.

## Role of funding source

This study was supported by the 10.13039/501100003478Ministry of Health, Labour and Welfare (No. 28131001 and No. 19GC1014). No funding body had any role in the study design, data collection and analysis, decision to publish, or manuscript preparation.

## Contributors

TM, AT, and YoK devised the study protocol. AT, TU, ST, YuK, and TM contributed to conducting the study and data collection. AT, KT, and AT contributed to data analysis. AT drafted the manuscript, and all authors contributed to the interpretation and critically reviewed it. All authors approved the final version of the manuscript.

## Author disclosure

None

## CRediT authorship contribution statement

**Ayumi Takano:** Writing – original draft, Visualization, Project administration, Methodology, Formal analysis, Data curation, Conceptualization. **Toshihiko Matsumoto:** Writing – review & editing, Supervision, Project administration, Methodology, Investigation, Funding acquisition, Data curation, Conceptualization. **Yousuke Kumakura:** Writing – review & editing, Project administration, Investigation, Data curation, Conceptualization. **Yuka Kanazawa:** Writing – review & editing, Project administration, Investigation, Data curation. **Shiori Tsutsumi:** Writing – review & editing, Project administration, Investigation, Data curation. **Takashi Usami:** Writing – review & editing, Project administration, Investigation, Data curation. **Tatsuhiko Anzai:** Writing – review & editing, Validation, Methodology, Formal analysis. **Kunihiko Takahashi:** Writing – review & editing, Validation, Methodology, Formal analysis.

## Declaration of Competing Interest

The authors declare that they have no known competing financial interests or personal relationships that could have appeared to influence the work reported in this paper.

## References

[bib1] Boeri M., Gibson D., Boshears P. (2014). Conceptualizing social recovery: recovery routes of methamphetamine users. J. Qual. Crim. Justice Criminol..

[bib2] Boeri M.W., Harbry L., Gibson D. (2009). A qualitative exploration of trajectories among suburban users of methamphetamine. J. Ethnogr. Qual. Res.

[bib3] Boeri M., Lamonica A., Harbry L. (2011). Social recovery, social capital, and drug courts. Pr. Anthr..

[bib4] Boshears P., Boeri M., Harbry L. (2011). Addiction and sociality: perspectives from methamphetamine users in suburban USA. Addict. Res. Theory.

[bib5] Brookfield S., Fitzgerald L., Selvey L., Maher L. (2019). Turning points, identity, and social capital: a meta-ethnography of methamphetamine recovery. Int. J. Drug Policy.

[bib6] Cumming C., Kinner S.A., McKetin R., Li I., Preen D. (2020). Methamphetamine use, health and criminal justice system outcomes: a systematic review. Drug Alcohol Rev..

[bib7] Degenhardt L., Hall W. (2012). Extent of illicit drug use and dependence, and their contribution to the global burden of disease. Lancet.

[bib8] Dobkin C., Nicosia N. (2009). The war on drugs: methamphetamine, public health, and crime. Am. Econ. Rev..

[bib9] Graves B.D., Fendrich M. (2024). Community-based substance use treatment programs for reentering justice-involved adults: a scoping review. Drug Alcohol Depend. Rep..

[bib10] Guerin A.A., Bridson T., Plapp H.M., Bedi G. (2023). A systematic review and meta-analysis of health, functional, and cognitive outcomes in young people who use methamphetamine. Neurosci. Biobehav Rev..

[bib11] Hazama K., Katsuta S. (2023). Factors to reduce drug-related recidivism among paroled methamphetamine users in Japan: 10-year data analysis. Int. J. Offender Ther. Comp. Criminol..

[bib12] Huang M.-C., Fang S.-C., Lin C., Lin T., Chang H.-M., Yang T.-W., Chen L.-Y. (2023). Risk factors for relapse among methamphetamine users receiving a joint legal–medical treatment program as a diversion intervention: a one-year follow-up study. J. Subst. Use Addict. Treat..

[bib13] Kato S. (2018). Probation in Japan: engaging the community. Ir. Probat. J..

[bib14] Kondo A., Shimane T., Takahashi M., Kobayashi M., Otomo M., Takeshita Y., Matsumoto T. (2023). Sex differences in the characteristics of stimulant offenders with a history of substance use disorder treatment. Neuropsychopharmacol. Rep..

[bib15] Liebregts N., Rigoni R., Petruželka B., Barták M., Rowicka M., Zurhold H., Schiffer K. (2022). Different phases of ATS use call for different interventions: a large qualitative study in Europe. Harm Reduct. J..

[bib16] Manja V., Nrusimha A., Gao Y., Sheikh A., McGovern M., Heidenreich P.A., Sandhu A.T.S., Asch S. (2023). Methamphetamine-associated heart failure: a systematic review of observational studies. Heart.

[bib17] Marshall B.D.L., Werb D. (2010). Health outcomes associated with methamphetamine use among young people: a systematic review. Addiction.

[bib18] Martens M.S., Zurhold H., Rosenkranz M., O’donnell A., Addison M., Spencer L., McGovern W., Gabrhelík R., Petruželka B., Rowicka M., Liebregts N., Degkwitz P., Kaner E., Verthein U. (2020). Using life course charts to assess and compare trajectories of amphetamine type stimulant consumption in different user groups: a cross-sectional study. Harm Reduct. J..

[bib19] Matsumoto, T., 2017. [Cohort study for people with drug use problems on probation: dissemination of the research project and investigation of the outcomes (2017)]. 〈https://www.ncnp.go.jp/nimh/yakubutsu/report/pdf/H29-2.pdf〉

[bib20] Matsumoto, T., 2019. [Cohort study for people with drug use problems on probation: dissemination of the research project and investigation of the outcomes (2019)]. 〈https://www.ncnp.go.jp/nimh/yakubutsu/report/pdf/R1-2.pdf〉

[bib21] Matsumoto, T., 2022. [Cohort study for people with drug use problems on probation: dissemination of the research project and investigation of the outcomes (2022)]. 〈https://www.ncnp.go.jp/nimh/yakubutsu/report/pdf/R4_matumoto.pdf〉

[bib22] McDonell M.G., Rawson R. (2023). Introduction to the special issue on stimulants. J. Subst. Use Addict. Treat..

[bib23] McKetin R., Gardner J., Baker A.L., Dawe S., Ali R., Voce A., Leach L.S., Lubman D.I. (2016). Correlates of transient versus persistent psychotic symptoms among dependent methamphetamine users. Psychiatry Res..

[bib24] O’Donnell A., Addison M., Spencer L., Zurhold H., Rosenkranz M., McGovern R., Gilvarry E., Martens M.S., Verthein U., Kaner E. (2019). Which individual, social and environmental influences shape key phases in the amphetamine type stimulant use trajectory? A systematic narrative review and thematic synthesis of the qualitative literature. Addiction.

[bib25] Research and Training Institute of the Ministry of Justice. 2023. White paper on crime 2023. Ministry of Justice. 〈https://www.moj.go.jp/content/001416537.pdf〉

[bib26] Sampson D., Heinsch M., Geddes J., Velleman R., Velleman G., Teesson M., Newton N., Kay-Lambkin F. (2023). I no longer know that person: experiences of families living with someone using crystal methamphetamine. PLoS One.

[bib27] Shimane T., Imamura A., Ikeda K., Yamamoto M., Tsuji M., Nagayo Y., Ohkubo T., Ohta M., Kanda H., Okazaki S., Ohe M., Matsumoto T. (2015). Reliability and validity of the Japanese version of the DAST-20. Nihon Arukoru Yakubutsu Igakkai Zasshi.

[bib28] Shoptaw S., Li M.J., Javanbakht M., Ragsdale A., Goodman-Meza D., Gorbach P.M. (2022). Frequency of reported methamphetamine use linked to prevalence of clinical conditions, sexual risk behaviors, and social adversity in diverse men who have sex with men in Los Angeles. Drug Alcohol Depend..

[bib29] Skinner H.A. (1982). The drug abuse screening test. Addict. Behav..

[bib30] Sommers I., Baskin D., Baskin-Sommers A. (2006). Methamphetamine use among young adults: health and social consequences. Addict. Behav..

[bib31] Tait R.J., Whetton S., Shanahan M., Cartwright K., Ferrante A., Gray D., Kaye S., McKetin R., Pidd K., Ritter A., Roche A., Allsop S. (2018). Quantifying the societal cost of methamphetamine use to Australia. Int J. Drug Policy.

[bib32] United Nations Office on Drugs and Crime. 2023. World Drug Report 2023. 〈https://www.unodc.org/unodc/en/data-and-analysis/world-drug-report-2023.html〉

[bib33] Vu N.T.T., Holt M., Phan H.T.T., Le H.T., La L.T., Tran G.M., Doan T.T., Nguyen T.N.N., de Wit J. (2016). Amphetamine-type stimulant use among men who have sex with men (MSM) in Vietnam: results from a socio-ecological, community-based study. Drug Alcohol Depend..

[bib34] Wang G., Shi J., Chen N., Xu L., Li J., Li P., Sun Y., Lu L. (2013). Effects of length of abstinence on decision-making and craving in methamphetamine abusers. PLoS One.

[bib35] Whiteford H.A., Degenhardt L., Rehm J., Baxter A.J., Ferrari A.J., Erskine H.E., Charlson F.J., Norman R.E., Flaxman A.D., Johns N., Burstein R., Murray C.J.L., Vos T. (2013). Global burden of disease attributable to mental and substance use disorders: findings from the Global Burden of Disease Study 2010. Lancet.

[bib36] Yamamoto T., Kimura T., Tamakoshi A., Matsumoto T. (2023). Biennial changes in the characteristics of patients with methamphetamine use disorder in Japan from 2000 to 2020. J. Psychoact. Drugs.

